# Challenging Surgical Management of Giant Uterine Leiomyomas With Ileal Resection: A Clinical Case Study

**DOI:** 10.7759/cureus.66017

**Published:** 2024-08-02

**Authors:** Pallavi Yadav, Manjusha Agrawal, Kamlesh Chaudhari, Arman Sindhu

**Affiliations:** 1 Obstetrics and Gynecology, Jawaharlal Nehru Medical College, Datta Meghe Institute of Higher Education and Research, Wardha, IND; 2 Respiratory Medicine, Jawaharlal Nehru Medical College, Datta Meghe Institute of Higher Education and Research, Wardha, IND

**Keywords:** anemia, bowel adhesion, hysterectomy, laparotomy, leiomyoma, uterine fibroids

## Abstract

Uterine fibroids, or leiomyomas, are common benign tumors of the uterus, generally asymptomatic but potentially causing severe symptoms and complications in some cases, as demonstrated in this report. This case presents significant management challenges due to the fibroids' size, number, and location, including an unusual complication involving adhesion to the ileum. A 40-year-old female with a history of P2L1D1 and no significant comorbidities presented with three months of progressive abdominal pain and a rapidly enlarging mass resembling a 30- to 32-week gravid uterus and heavy menstrual bleeding. Clinical findings included severe anemia with a hemoglobin level of 5.5 g/dL. Imaging studies revealed a bulky uterus with numerous multilobulated, well-defined, solid, hypoechoic fibroids subserosally and intramurally, raising suspicions of sarcomatous conversion. The patient underwent a laparotomy, which involved the resection of multiple large subserosal fibroids and a total abdominal hysterectomy, necessitated by extensive uterine distortion and the patient's preference against fertility preservation. A significant intraoperative discovery was the adhesion of fibroids to the ileum, which required bowel resection and anastomosis. This case emphasizes the complexity of managing extensive uterine fibroids, highlighting the need for thorough preoperative assessment, preparation for potential intraoperative complications, and the importance of a multidisciplinary surgical approach. The successful management and uneventful recovery underscore the effectiveness of proactive and comprehensive surgical intervention in cases with significant fibroid burden and associated anatomical challenges.

## Introduction

Uterine fibroids, also known as leiomyomas, are the most common benign pelvic tumors in women of reproductive age. Epidemiological studies estimate that fibroids affect up to 70%-80% of women by age 50 [1]. They are monoclonal tumors arising from the smooth muscle cells of the uterus and can vary widely in size, number, and location within the uterine tissue [2]. The clinical presentation of fibroids can range from being entirely asymptomatic to causing severe symptoms that significantly impact the patient's quality of life. Common symptoms include abnormal uterine bleeding, notable menorrhagia, pelvic pressure or pain, and, in some cases, reproductive dysfunction, which may manifest as infertility or recurrent miscarriages [3]. The growth of fibroids is believed to be influenced by hormonal factors, primarily estrogen and progesterone, as they tend to increase in size during reproductive years and regress after menopause [4].

Complications from fibroids, while less common, can be severe. These include acute pain due to fibroid degeneration, renal hydronephrosis caused by pressure on the ureters, and rare but potentially life-threatening conditions such as malignant transformation into leiomyosarcoma [5]. Additionally, large fibroids can lead to significant anatomical distortions with potential implications on adjacent organs, as highlighted in cases where bowel adhesions occur, complicating surgical management [6]. Surgical treatment remains a mainstay for symptomatic fibroids, particularly in cases where fibroid size and symptomatology impair normal function. Options range from conservative myomectomy, preserving the uterus and fertility, to total hysterectomy, which is definitive but precludes future fertility. Decision-making in surgical management depends on the patient's age, symptom severity, fibroid characteristics, and reproductive desires [7]. This case illustrates the complexity of diagnosing and managing a patient with extensive symptomatic fibroids, compounded by a rare complication of ileal adhesion, which necessitated a multidisciplinary approach to surgical intervention.

## Case presentation

A 40-year-old female with an obstetric history of P2L1D1, having experienced both vaginal deliveries, presented at the outpatient department of our tertiary care hospital. She reported a three-month history of progressive abdominal pain and a noticeably enlarging mass in her abdomen, which she described as having grown to the size of a 30- to 32-week gravid uterus during this period. Accompanying these symptoms was heavy menstrual bleeding, although she maintained a regular menstrual cycle lasting 5-6 days every 28-30 days. The patient had no significant past medical history, including common comorbidities such as hypertension, diabetes, or thyroid disorders. Additionally, she did not report any nausea, vomiting, or loss of appetite.

**Figure 1:**
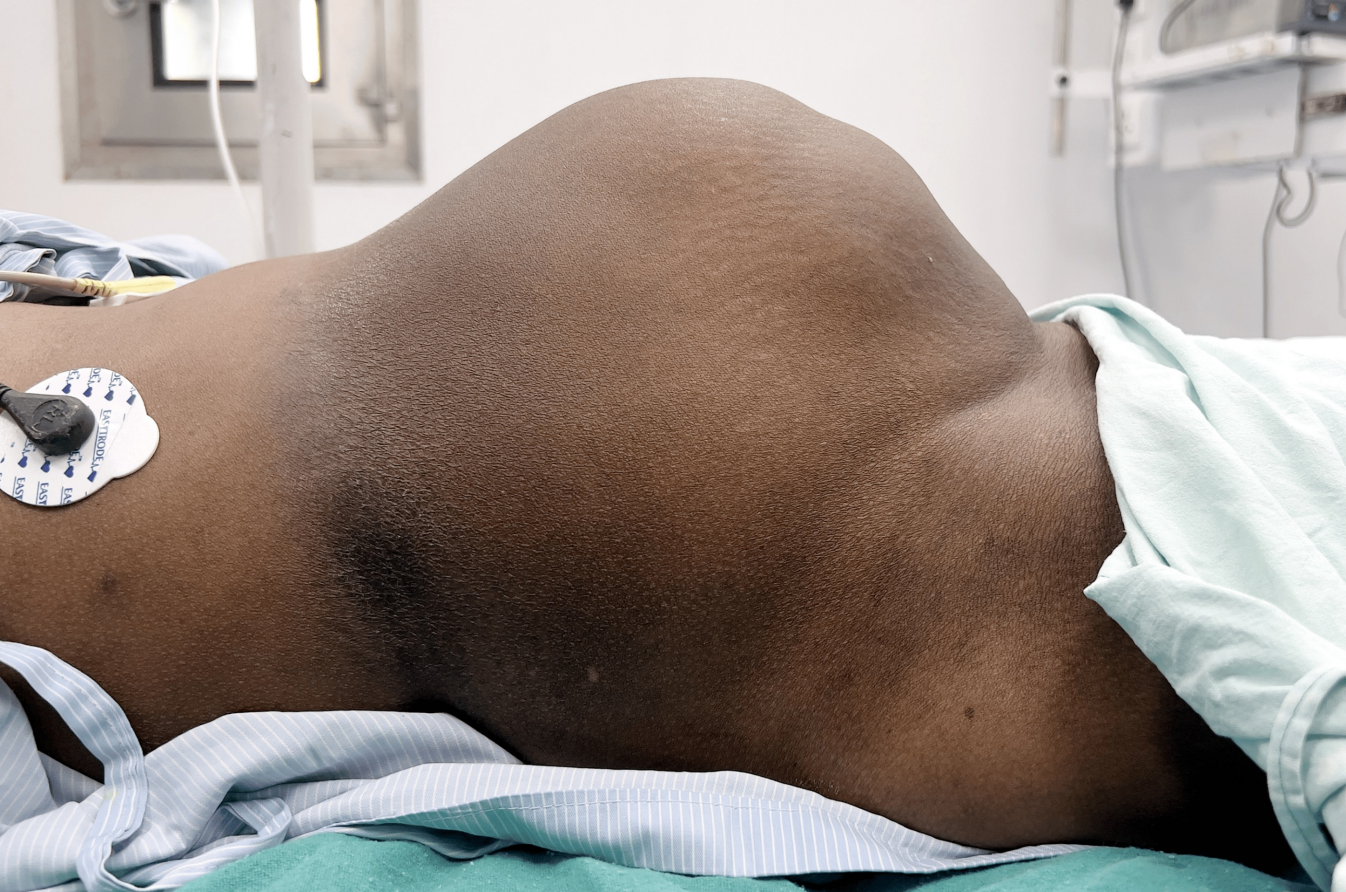
Severely anemic patient who presented with a large, visible abdominal mass

Upon physical examination, the patient appeared severely anemic and presented with a large, visible abdominal mass (Figure 1). The mass was firm and non-tender, with multiple masses palpable upon further assessment. A pelvic examination revealed a posteriorly displaced cervix with no lesions or signs of inflammation. A PAP smear performed at this time returned normal results. Laboratory tests confirmed severe anemia with a hemoglobin level of 5.5 g/dL, but other investigations, including tumor markers, showed normal results.

Imaging studies were critical in assessing the extent of the patient's condition. An ultrasound of the abdomen revealed a bulky uterus with multiple multilobulated, well-defined, solid, hypoechoic soft tissue mass lesions subserosally and intramurally, with the most extensive lesions measuring up to 127 × 101 mm. These findings extended from the pelvis to the epigastric region. A subsequent CT scan supported these findings, depicting a bulky uterus with multiple varying-sized uterine fibroids scattered throughout the myometrium and multiple heterogenous soft tissue masses predominantly in the bilateral lumbar and umbilical regions, some arising from the uterine wall, raising suspicion of sarcomatous conversion.

**Figure 2:**
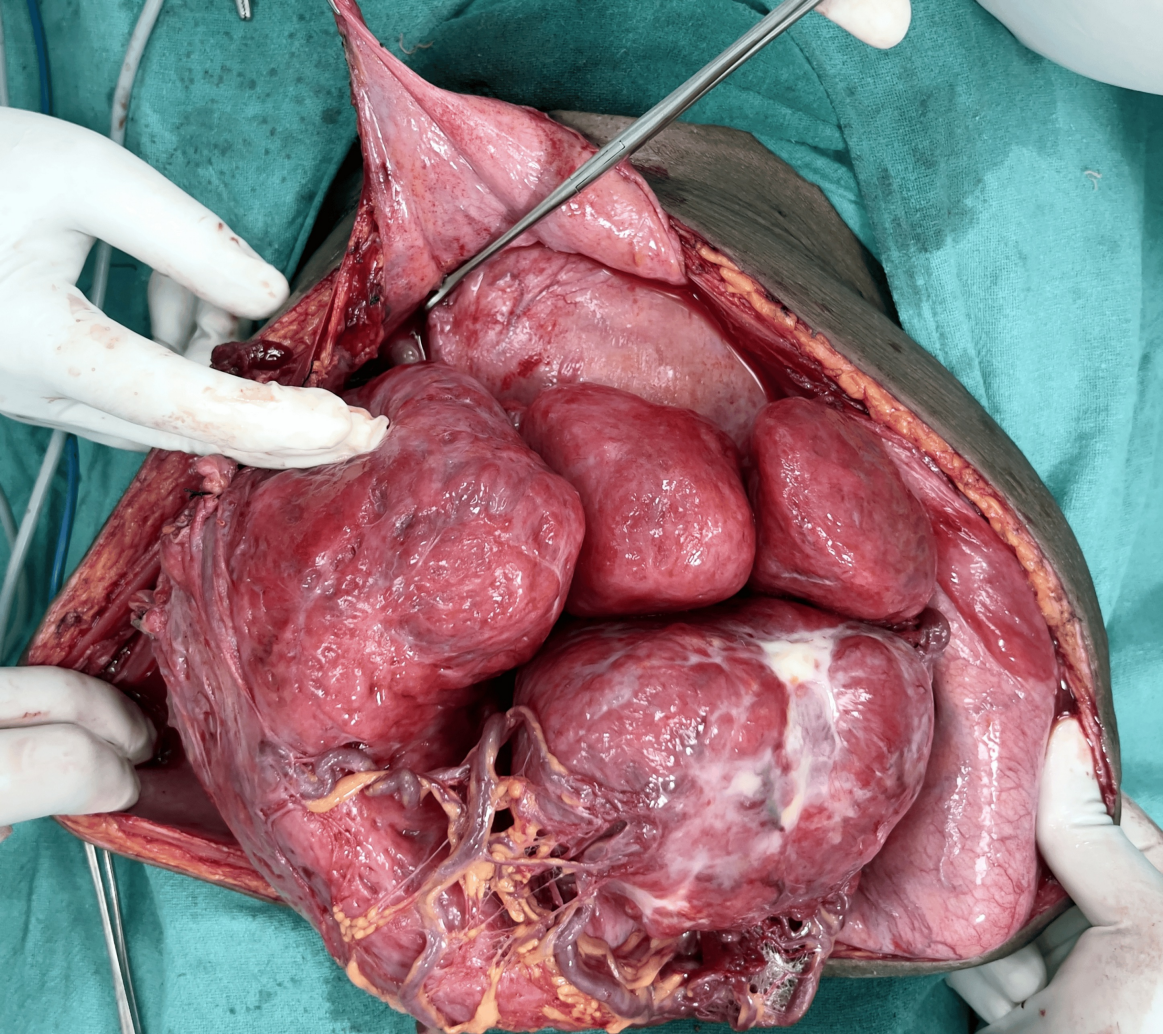
Benign leiomyoma

Given the severity of anemia and the extensive nature of the fibroids, the patient underwent a transfusion with five bags of packed cells before surgery. A laparotomy was then performed, revealing multiple large subserosal fibroids arising from the anterior surface of the uterus. During surgery, it was discovered that a segment of the ileum, approximately 20 cm proximal, had adhered to one of the fibroids, necessitating a resection and double-layer side-to-side anastomosis. Intraoperative frozen section analysis of the resected fibroids indicated benign leiomyoma (Figure 2). Considering the patient's preference not to preserve fertility and the extensive distortion of the uterine structure by the tumor mass, a total abdominal hysterectomy was performed. Care was taken to protect the ureters during the procedure.

**Figure 3:**
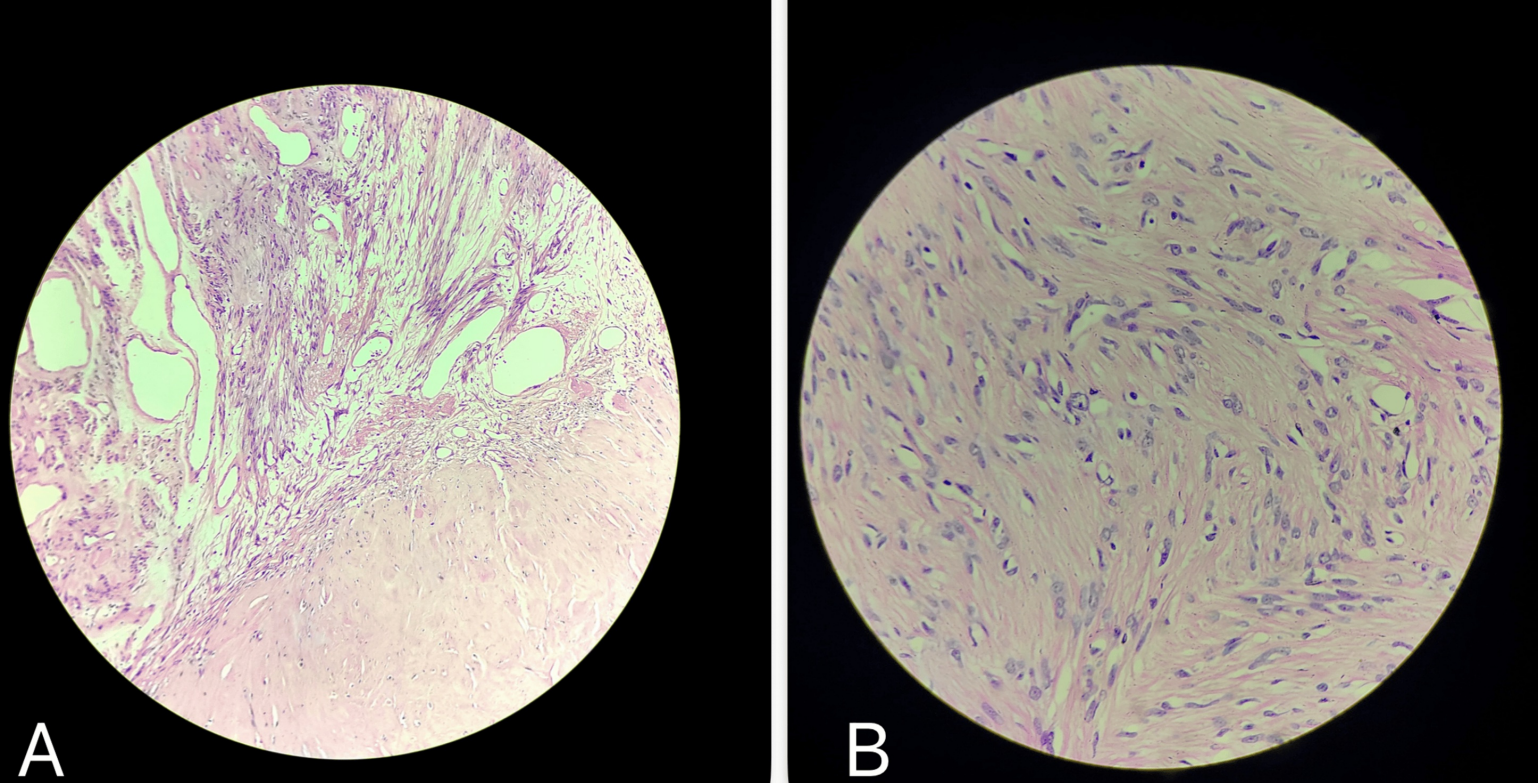
Histopathological examination showed (A) leiomyoma with hyaline changes and (B) leiomyoma with ancient changes (hematoxylin and eosin, 40× magnification)

Histopathological examination of the resected uterus postoperatively confirmed benign leiomyoma with ancient and hyaline changes (Figure 3).

The patient's postoperative period was uneventful, and she was discharged on the eighth day post-surgery. This case highlights the challenges and complexities of diagnosing and managing extensive uterine fibroids, especially when compounded by unexpected surgical findings such as bowel adhesion.

## Discussion

In this case, the patient presented with symptoms typical of uterine fibroids, including abdominal pain, heavy menstrual bleeding, and a rapidly enlarging abdominal mass. Diagnostic imaging, including ultrasound and CT scan, played a crucial role in confirming the diagnosis and assessing the extent of fibroid involvement. Ultrasound is often the initial imaging modality of choice due to its accessibility and ability to differentiate fibroids from other pelvic masses [8]. CT scan provides additional information about fibroids' size, location, and characteristics, as well as potential complications such as sarcomatous transformation, as seen in this case [9].

Surgical management remains the mainstay of treatment for symptomatic fibroids, with options ranging from myomectomy to hysterectomy, depending on the patient's desire for fertility preservation and the extent of the disease. In this case, the decision for total abdominal hysterectomy was made due to the patient's severe symptoms, desire to avoid future pregnancies, and the extensive nature of the fibroids, which distorted the uterine anatomy. Intraoperative findings, including bowel adhesion to the fibroids, added complexity to the surgical procedure, highlighting the importance of thorough preoperative evaluation and intraoperative vigilance. Bowel involvement by fibroids is a rare but recognized complication, occurring in less than 1% of cases [10]. In cases of bowel adhesion, careful dissection and, if necessary, bowel resection may be required to achieve complete fibroid removal and prevent postoperative complications.

Histopathological examination of the resected specimen confirmed the diagnosis of benign leiomyoma with ancient and hyaline changes. While most fibroids are benign, sarcomatous transformation is a rare but severe complication, with an estimated incidence of 0.1-0.5% [11]. Early recognition of suspicious features on imaging and intraoperative frozen section analysis is crucial for guiding surgical management and optimizing patient outcomes. This case underscores the challenges and complexities involved in the diagnosis and management of extensive uterine fibroids. A multidisciplinary approach, including gynecologists, radiologists, and general surgeons, is essential for comprehensive evaluation and treatment planning. Patient education and shared decision-making are integral to ensuring optimal outcomes and patient satisfaction.

## Conclusions

In conclusion, the management of extensive uterine fibroids, in this case, exemplifies the complexity and challenges often encountered in clinical practice. The patient's symptoms were effectively addressed through a combination of thorough diagnostic evaluation, including imaging studies and intraoperative assessment, along with multidisciplinary collaboration. Surgical intervention, including total abdominal hysterectomy and bowel resection with anastomosis, was necessary to alleviate the patient's severe symptoms and prevent potential complications associated with the fibroid pathology. Histopathological examination confirmed benign leiomyoma, underscoring the importance of accurate diagnosis and appropriate surgical planning. This case highlights the importance of individualized treatment approaches tailored to the patient's clinical presentation, preferences, and the extent of fibroid disease, ultimately leading to a successful outcome and improved quality of life for the patient.
